# Knowledge/perception and attitude/practices of populations of two first-line communities of the Centre Region of Cameroon regarding onchocerciasis and black fly nuisance and bio-ecology

**DOI:** 10.1186/s13071-021-05048-y

**Published:** 2021-10-23

**Authors:** André Domche, Hugues C. Nana-Djeunga, Linda Djune Yemeli, Cédric Lenou Nanga, Michel Boussinesq, Flobert Njiokou, Sébastien Pion, Joseph Kamgno

**Affiliations:** 1 Centre for Research on Filariasis and Other Tropical Diseases (CRFilMT), Yaoundé, Cameroon; 2 Parasitology and Ecology Laboratory, Department of Animal Biology and Physiology, Faculty of Science, University of Yaoundé 1, Yaoundé, Cameroon; 3 Molecular Diagnosis Research Group, Biotechnology Centre of the University of Yaoundé 1 (BTC-UY I), Yaoundé, Cameroon; 4 Institut de Recherche pour le Développement (IRD), UMI 233- INSERM U1175-Montpellier University, Montpellier, France; 5 Department of Public Health, Faculty of Medicine and Biomedical Sciences, University of Yaoundé 1, Yaoundé, Cameroon

**Keywords:** Knowledge, Attitude, Practice, Onchocerciasis, Black fly, Vector control

## Abstract

**Background:**

Despite high black fly densities in persisting onchocerciasis foci in Cameroon, no vector control has ever been carried out to complement Community-Directed Treatment with Ivermectin (CDTI). As a prelude to community-based vector control, this study explored knowledge/perception and attitude/practice (KAP) of populations of two first-line communities regarding onchocerciasis, black fly nuisance and bio-ecology.

**Methods:**

A cross-sectional survey was conducted in two communities of the Bafia Health District, following a household-based interview approach using a structured questionnaire. KAP scores were calculated and categorised as good or poor based on the number of correct or positive responses. Associations between KAP and socio-demographic parameters were explored using logistic regression models.

**Results:**

A total of 215 individuals aged 15–100 years were interviewed. Positive associations were observed between good knowledge/perception and age and the duration of residency in the community. Most respondents (91.6%) described having post-biting sequels (oedema, itching) and more than half (69.3%) admitted that black fly bites affect their productivity. Although 81.4% of the respondents stated that black fly densities are higher during the rainy season, only 10.7% of them knew that they breed in the river. Also, 59.5% of the interviewees stated that black flies bite not only outdoors but also indoors, and 78.6% of enrolees were ready to help to fight against black flies. Most of the respondents were well aware of onchocerciasis, even though the transmission mode and vector bio-ecology were not well known.

**Conclusion:**

This study revealed the need to implement community-based vector control to support CDTI in the fight against onchocerciasis and to reduce black fly nuisance.

**Graphical abstract:**

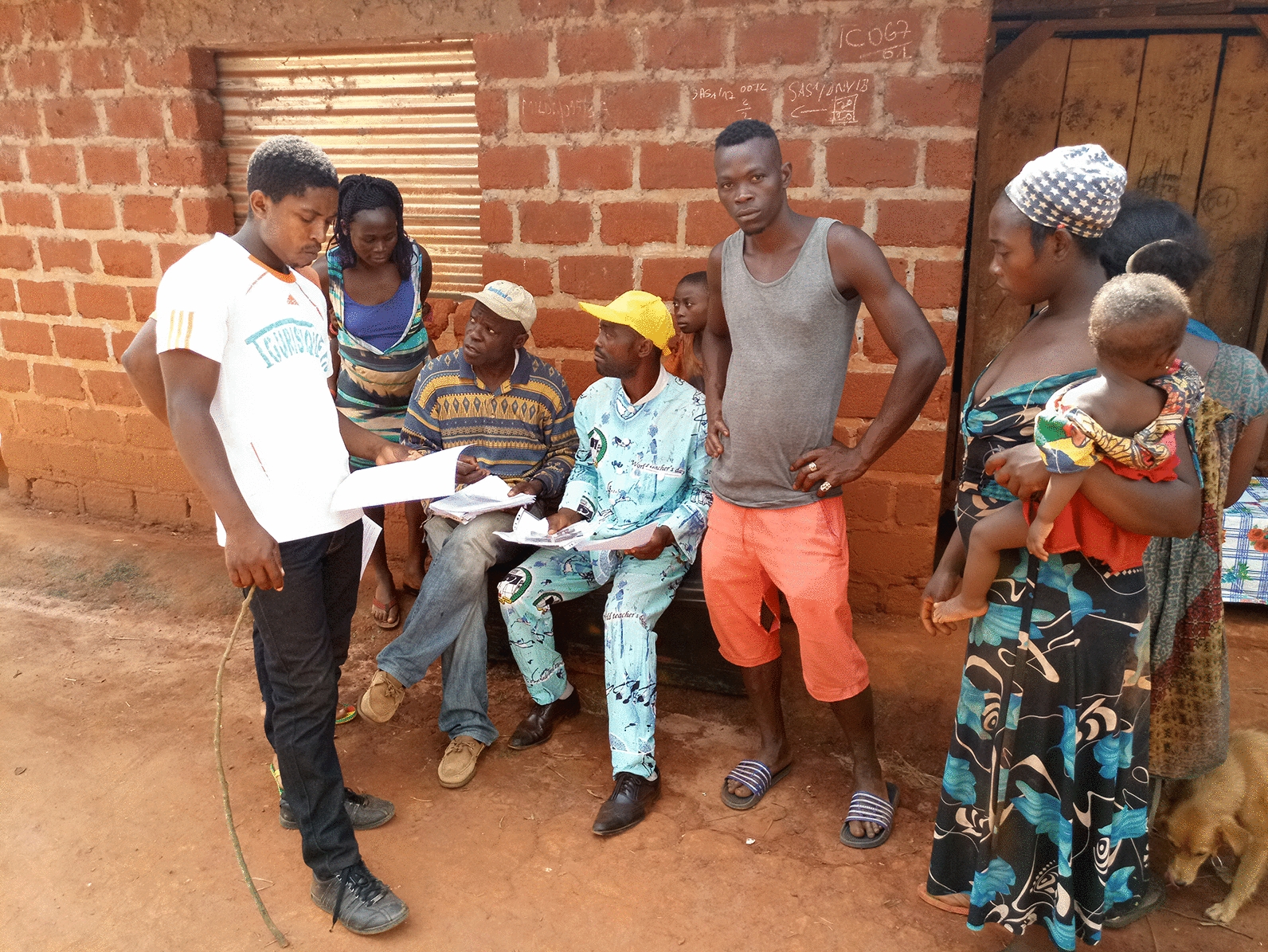

**Supplementary Information:**

The online version contains supplementary material available at 10.1186/s13071-021-05048-y.

## Background

Onchocerciasis is a debilitating vector-borne disease caused by the nematode parasite *Onchocerca volvulus* and transmitted by black flies of the genus *Simulium*. Onchocerciasis is currently endemic in 31 African countries, 2 Latin American countries and Yemen. According to latest estimates, about 17 million people are infected with *O. volvulus* [1]. The Centre Region of Cameroon, especially the Mbam valley, belongs to the forest-savannah transition zone where the repercussions of the disease have been among the highest in the country, with initial microfilaridermia prevalences ranging from 68 to 99% and community microfilarial load > 100 microfilariae/skin snip (mf/ss) in some villages prior the commencement of interventions [2]. In this area, onchocerciasis was also associated with high prevalence of blindness and epilepsy [2, 3] as well as with excess mortality associated with both conditions [4, 5].

To fight against onchocerciasis, two main programmes have been set up in Africa. The Onchocerciasis Control Programme in West Africa (OCP), launched in 1975, was the first and focused on vector control through weekly aerial application of insecticides to eliminate black fly larvae, reduce adult black fly population density to zero and interrupt transmission of *O. volvulus* [6–8]. After a very successful implementation in 11 West African countries, the programme ended in 2002. Subsequently, the African Programme for Onchocerciasis Control (APOC) was launched in 1995 and, unlike the OCP strategy, focused on preventive chemotherapy through the Community-Directed Treatment with Ivermectin (CDTI) strategy, which is based on annual or multi-annual mass administration of ivermectin in meso- and hyper-endemic communities [9]. As part of APOC, vector control activities were restricted to the island of Bioko (Equatorial Guinea) and isolated foci in Uganda and Tanzania [10].

CDTI was launched in the Mbam Valley focus in 1998. Parasitological surveys conducted in 2015 revealed that onchocerciasis is persisting in that focus, with microfilarodermia prevalence > 50% in some communities despite > 20 years of CDTI [11]. A subsequent entomological study conducted in 2016/2017 reported very high indicators, with annual biting rates (ABR) up to 606,370 bites/person/year and annual transmission potentials (ATP) of 4488 infectious larvae/person/year at some points on the bank of the Mbam River [12]. This high level of transmission, related to high black fly densities, might be the main driving factor explaining the persistence of onchocerciasis in this area. However, despite the abundance of breeding sites and the very high black fly densities, no large-scale vector control has ever been carried out to complement CDTI.

From previous experience in Brazil and Uganda, community involvement in actions related to black fly control could increase their effectiveness and sustainability [13–15]. As a prelude to the implementation of a pilot community-based strategy for black fly control in the Mbam Valley, the present study aimed to investigate the knowledge and perception of the populations regarding onchocerciasis, black fly nuisance and bio-ecology to optimise the control strategy.

## Methods

### Study area and population

The study was conducted in the Bafia Health District (4°44'23.748''N, 11°14'0.024''E), located in the Mbam-et-Inoubou Division, Centre Region, about 120 km north of Yaoundé, the political capital of Cameroon. In this Health District, latest estimates revealed that onchocerciasis microfilaridermia prevalence ranged from 24.4 to 57.0% [11]. It is a forest-savannah transition zone irrigated by an important network of fast-flowing rivers, notably the Sanaga and its main tributary, the Mbam River, which in its course presents several series of falls and rapids and many substrata favourable to *Simulium damnosum* (sensu lata) breeding. Study conducted by Hendy and colleagues in 2015–2016 revealed the presence of *S. squamosum* and *S. mengense* in this area [12]. Two first-line villages (communities closest to the river where the black fly breeding sites are located), namely Bayomen (N4.86499, E11.10804) and Biatsota (N4.77640, E11.28884) (see Additional file 1: Figure S1), targeted for the pilot community-based vector control, were selected for the current study. These communities were selected because of the persistence of onchocerciasis despite > 20 years of CDTI and the high vector densities previously recorded. In 2017, the population of the Bafia Health District was estimated at about 161,400 inhabitants, with 388 residents in Biatsota and 481 in Bayomen [16]. The main activities of this population are agriculture (mainly subsistence cocoa and food crops), fishing and sand mining in the Mbam River.

### Study design and data collection

A cross-sectional survey was conducted in August 2020 in the two selected communities. All individuals living in the targeted communities at the time of survey, aged ≥ 15 years, who provided their oral consent to participate in the study (consents were obtained from parent or legal guardian for those aged < 18 years old) and were able to understand and answer questions in French, or in their local language (Bafia), were eligible for the study. A door-to-door systematic survey was conducted in each community. All households of each community were visited and all individuals at home at the time of the visit and meeting the inclusion criteria were interviewed.

Information on the participants’ knowledge and perception of onchocerciasis and black fly bio-ecology and nuisance was captured using a structured questionnaire (Additional file 2: Table S1) with multiple choice questions. After explaining the objectives and schedule of the study to participants, a single interviewer with a local guide administered the questionnaire to the volunteers individually to avoid any influence of other household members on their answers. Although onchocerciasis is the most prevalent filariasis in the study area, the term “manonomanbou” (meaning onchocerciasis in Bafia) was used to avoid confusion. Local terms and expressions such as “mbou” for black fly were also used, and questions were reformulated if needed to ensure a better understanding of the respondents. The main items captured were (i) the socio-demographic characteristics (gender, age, profession, village of residence and duration of residency in the village), (ii) knowledge and perception of interviewees regarding onchocerciasis, black fly vectors, biting place (indoor or outdoor), seasonal variation and breeding sites and (iii) attitudes/practices of enrolees related to black fly biting and nuisance. The occupations of interviewees were organised into two categories: those predisposed to high exposure to black fly bites (farming, sand mining and fishing) and those predisposed to low exposure to black fly bites (other occupation groups such as housewife, trader, etc.).

### Sample size

Since there was no previous information on the level of community knowledge and perception regarding onchocerciasis, black fly nuisance and bio-ecology in the present study area, we hypothesised that at least 50% of the target population (i.e. residents ≥ 15 years of age) would have a good level of knowledge about the disease and black fly bio-ecology. Hence, sample size was estimated taking this as the starting point with 95% confidence level and 5% margin of error, resulting in the final sample size of 384.

### Statistical analysis

All data collected were recorded into a purpose-built Microsoft Excel database and analysed using GraphPad Prism version 8. Categorical variables (gender, occupation, knowledge, etc.) were summarised using proportions with 95% confidence interval, and continuous variables (age, duration of residency in the community, KAP score) were described using median and interquartile range (IQR).

To assess the overall knowledge of respondents, the following questions were asked of the enrolees: (i) Have you ever heard of onchocerciasis? (ii) Do you know the transmission mode? (iii) Have you ever heard of black flies? (iv) Do you know what disease they transmit? (v) During which period of the year are you most bitten by black flies? (vi) During which period are black flies more abundant? (vii) Do you know where black flies breed? (viii) Do you know about Mectizan®? Response to each question was attributed a score (“1” for Yes or a right answer and “0” for No or an incorrect answer) and an individual KAP score generated by summing up the scores of the eight questions (the maximum score possible was 8). Moreover, respondent’s knowledge was organised in two categories: (i) poor knowledge when the individual KAP score was lower than the average KAP score regardless of community of residency of participants; (ii) good knowledge when the individual KAP score was higher than or equal to the average KAP score regardless of participants' community of residency.

Chi-square test was used to compare the knowledge among age, gender and time of residency in the community. The association between knowledge and the different covariates was computed using binary logistic regression models, expressed with odds ratio (OR) and its 95% confidence interval. A 5% threshold for significance was considered for all analyses.

## Results

### Socio demographic characteristics of the surveyed population

A total of 215 individuals (132 in Bayomen and 83 in Biatsota) aged to 15–100 years (median: 33 years, IQR: 22–50 years) were interviewed. Tables 1 and 2 summarise the socio-demographic characteristics and the knowledge/perception and attitudes/practices of respondents, respectively. In the two communities, most of the respondents were female (53.0% in Bayomen and 54.2% in Biatsota); 70.7% of the respondents were engaged in occupations with a high predisposing risk of exposure (farming, sand mining and fishing). The duration of residency in the community ranged from 3 months to 100 years (median: 17 years, IQR: 5–33 years) with 29.3% of the respondents having always lived in the villages.

**Table 1:** Socio-demographic characteristics of the surveyed individuals

Variables	No. individuals in Bayomen (%)	No. individuals in Biatsota	Total no. individuals visited (%)
Numbers	132	83	215
Age			
[15–25]	47 (35.6)	25 (30.1)	72 (33.5)
[26–35]	33 (25.0)	12 (14.5)	45 (20.9)
[36–45]	23 (17.4)	15 (18.1)	38 (17.7)
[46–55]	15 (11.4)	11 (13.3)	26 (12.1)
> 55	14 (10.6)	20 (24.1)	34 (15.8)
Sex			
Male	62 (47.0)	38 (45.8)	100 (46.5)
Female	70 (53.0)	45 (54.2)	115 (53.5)
Occupation			
High-risk occupation	93 (70.5)	59 (71.1)	152 (70.7)
Low-risk occupation	39 (29.5)	24 (28.9)	63 (29.3)
Duration of residency			
< 1	4 (3.0)	2 (2.4)	6 (2.8)
[1–3]	31 (23.5)	8 (9.6)	39 (18.1)
[4–6]	13 (9.8)	3 (3.6)	16 (7.4)
[7–9]	11 (8.3)	1 (1.2)	12 (5.6)
> 10	73 (55.3)	69 (83.1)	142 (66.0)

*No.* number of

**Table 2:** Knowledge of the respondents on onchocerciasis and black fly bio-ecology in the targeted communities

Indicative questions	Response categories	Bayomen (%)	Biatsota (%)	Both villages (%)
Numbers		132	83	215
Have you ever heard about onchocerciasis?	Yes	119 (90.2)	71 (85.5)	190 (88.4)
	No	13 (9.8)	12 (14.5)	25 (11.6)
If “yes”, do you know transmission mode?	Black fly bites	23 (17.4)	27 (32.5)	50 (23.3)
	Mosquitoes’ bites	9 (6.8)	5 (6.0)	14 (6.5)
	Dirty food/water	1 (0.8)	0 (0)	1 (0.5)
	Don't know	86 (65.2)	39 (47.0)	125 (58.1)
Have you ever heard of black flies?	Yes	131 (99.2)	82 (98.8)	213 (99.1)
	No	1 (0.8)	1 (1.2)	2 (0.9)
Do you know that black flies can transmit diseases?	Yes	106 (80.3)	64 (77.1)	170 (79.1)
	No	26 (19.7)	19 (22.9)	45 (20.9)
If “yes”, which one?^a^	Filariasis	53 (40.2)	43 (51.8)	96 (44.7)
	Malaria	25 (18.9)	16 (19.3)	41 (19.1)
	Sleeping sickness	2 (1.5)	0 (0)	2 (0.9)
	Scabies	8 (6.1)	6 (7.2)	14 (6.5)
	Don't know	27 (20.5)	7 (8.4)	34 (15.8)
Do you have post-bite sequelae?	Yes	118 (89.4)	79 (95.2)	197 (91.6)
	No	14 (10.6)	4 (4.8)	18 (8.4)
If “yes”, what kind of effects?^a^	Itching	97 (73.5)	75 (90.4)	172 (80.0)
	Swelling	31 (23.5)	48 (57.8)	79 (36.7)
	Pain	6 (4.5)	2 (2.4)	8 (3.7)
Have you ever stopped working because of black fly bites?	Yes	95 (72)	54 (65.1)	149 (69.3)
	No	37 (28)	29 (34.9)	66 (30.7)
Do black flies also bite you at home?	Yes	127 (96.2)	83 (100)	210 (97.7)
	No	5 (3.8)	0 (0)	5 (2.3)
If “yes”, indoor or outdoor?	Indoor only	1 (0.75)	0 (0)	1 (0.5)
	Outdoor only	42 (31.8)	40 (48.2)	82 (38.1)
	Indoor and outdoor	84 (63.6)	43 (51.8)	127 (59.1)
How do you protect yourself from black fly bites?^a^	Clothes covering the body	97 (73.5)	74 (89.2)	171 (79.5)
	Palm oil	7 (5.3)	8 (9.6)	15 (7.0)
	Diesel	2 (1.5)	3 (1.5)	5 (2.3)
	Lemon extract	3 (2.3)	1 (1.2)	4 (1.9)
	No protection	28 (21.2)	4 (4.8)	32 (14.9)
During which period are black flies more abundant?	Rainy season	100 (75.8)	75 (90.4)	175 (81.4)
	Dry season	10 (7.6)	2 (2.4)	12 (5.6)
	All seasons	10 (7.6)	4 (4.8)	14 (6.5)
	Don’t know	12 (9.1)	2 (2.4)	14 (6.5)
Where do black flies lay their eggs?^a^	River	9 (6.8)	14 (16.9)	23 (10.7)
	Bush	36 (27.3)	9 (10.8)	45 (21.0)
	Dirty water	0 (0)	1 (1.2)	1 (0.5)
	Grass	1 (0.8)	2 (2.4)	3 (1.4)
	Don’t know	87 (65.9)	60 (72.3)	147 (68.4)
Would you be willing to help in the fight against black flies?	Yes	86 (65.2)	83 (100)	169 (78.6)
	No	43 (32.6)	0 (0)	43 (20.0)
	Don’t know	3 (1.4)	0 (0)	3 (1.4)
Do you know about Mectizan?	Yes	123 (93.2)	83 (100)	206 (95.8)
	No	9 (6.8)	0 (0)	9 (4.2)
Do you usually take Mectizan?	Yes	113 (85.6)	78 (94)	191 (88.3)
	No	18 (13.6)	05 (6)	23 (10.7)

^a^Some participants provided several answers

### Knowledge/perception and attitude/practices of the respondents regarding onchocerciasis and its black fly vectors

Most of the respondents (88.4%) acknowledged that they had already heard about onchocerciasis; the details of the 11.6% who had never heard about onchocerciasis are provided as Additional file 3: Table S2. Unlike onchocerciasis, almost all the interviewees (99.1%) acknowledged that they had already heard about black flies, though only 23.3% of them identified the latter as the vector of onchocerciasis. The majority of the study participants (91.6%) stated that they presented with sequelae when bitten by black flies, and more than half (69.3%) acknowledged having interrupted their work several times because of these bites. Itching/scratching (80.0%) and rashes (36.7%) were the most reported consequences of black fly bites. A total of 81.4% of the respondents considered that black fly densities are higher during the rainy season (mainly in September and October), and only 10.7% of survey participants knew that black flies breed in the river while 20.9% believed that they breed in the bush. More than half (59.5%) of the respondents stated that black flies bite not only outdoors but also indoors. As preventive methods against black fly bites, 80.0% of the respondents declared that they wear clothes as well as fabric gloves and socks that cover their entire body. Some respondents (8.4%) use topical application of plant extracts as repellents (7.0% use palm oil and 1.4% use lemon juice) while 2.8% use chemical products such as gas oil. Most (78.6%) of the interviewees declared that they would be willing to help in the fight against black flies (50.3% of the females and 49.7% of the males) if an effective and feasible strategy was proposed to them; 95.3% of respondents were familiar with ivermectin and 88.8% took it regularly during mass treatment campaigns.

### Association between knowledge regarding onchocerciasis and its black fly vectors and socio-demographic characteristics of the respondents

The scores of respondents ranged from 0 to 8 (median: 4; IQR: 4–6). For Biatsota, KAP scores ranged from 2 to 8 (median: 4; IQR: 4–7) while in Bayomen KAP scores ranged from 0 to 8 (median: 4; IQR: 4–6). Of the 215 individuals interviewed, 93 (43.3%) had good knowledge regarding onchocerciasis and black fly bio-ecology. The proportion of individuals having good knowledge/perception and attitude/practices was lower in Bayomen (39.4%) compared to Biatsota (68.9%), but not significantly different between the two communities (*P*-value: 0.3696).

The proportion of males (48.0%) exhibiting good knowledge/perception and good attitudes/practices seemed to be larger than that of females (39.1%), but the difference was not significant (*P*-value: 0.4107). Regarding the relationship between occupation and knowledge/perception or attitudes/practices, 45.4% of individuals practicing high-exposure activities had good knowledge compared to 38.1% of individuals practicing low-risk activities; no significant difference was found when comparing these proportions (*P*-value: 0.5315).

Univariate binary logistic regression to assess the association between knowledge and different covariates revealed a significant association between age and knowledge (OR: 1.0224, 95% CI 1.0055–1.0397; *P*-value: 0.0082) and between time of residency and knowledge (OR: 1.0155, 95% CI 1.0010–1.0303; *P*-value: 0.0336), with older people and those who had lived the longest in communities exhibiting the best knowledge/perception. Overall multivariate logistic regression revealed no significant differences between knowledge/perception about onchocerciasis, black fly nuisance and bio-ecology and socio-demographic parameters (*P*-value: 0.0984.) (Table 3).

**Table 3:** Multivariate logistic regression between KAP score and individual covariates

Variable	Odds ratio	95% CI	*P*-value
Age	1.0179	(0.994–1.0422)	0.1413
Sex	0.7967	(0.454–1.3960)	0.4271
Occupation	1.0222	(0.531–1.9654)	0.9476
Time of residence	1.0039	(0.984–1.0238)	0.7006
Constant			0.2707

### Association between knowledge regarding onchocerciasis/black fly bio-ecology and practices related to onchocerciasis control/prevention

A significant association was found between having already heard about onchocerciasis and its transmission mode (*P*-value < 0.0001). Similarly, there was a significant association between sequelae post *Simulium* bite (itching, swelling, pain) and the use of any of the above-mentioned protection means (clothes covering the body, palm oil, diesel, lemon extract) (*P*-value < 0.0001). Contrarily, there was no significant association between knowledge regarding onchocerciasis/black flies and adherence to ivermectin mass drug administration (*P*-value: 0.5788). Additionally, no association was found between stopping work because of black fly bites and the willingness to participate in vector control activities.

## Discussion

This survey was carried out in prelude to the implementation of a community-based strategy for black fly control and aimed to investigate the local populations' knowledge and perceptions vis-à-vis black fly nuisance and bio-ecology as well as their willingness to contribute to the fight against these vectors.

Most of the participants had already heard about onchocerciasis (this information has already been recorded in different foci in Africa [17, 18]), which could be because of the high endemicity level and important burden of the disease as well as annual CDTI (with social mobilisation and sensitisation of populations) implemented for > 20 years in the area. It was surprising that a relatively important proportion of interviewees declared that they had never heard about onchocerciasis, and in-depth investigation of their profiles revealed that these were mostly people who had spent > 10 years in the villages (Additional file 2: Table S2), which might suggest that the sensitisation strategy used by the community drug distributors (CDDs) is having some difficulty reaching its entire target. Positive associations were found between good knowledge/perception and age and duration of residency in the targeted communities; older individuals who had lived the longest in the villages had the best knowledge/perceptions. This might be explained by the fact that Bafia Health District is a historic focus of onchocerciasis with an important burden for populations, and door-to-door sensitisation campaigns are carried out by CDDs before each yearly treatment campaign. As in previous studies conducted in Ethiopia and Tanzania [19, 20], less than a quarter of the interviewees understood the link between onchocerciasis and black flies in Mbam Valley. Although most respondents had already heard about onchocerciasis, a range of misconceptions was also observed regarding the transmission mode (even mosquito bites were cited). Most of the respondents complained about itching just after black fly bites. This is because when black flies bite, they inject saliva containing a pruritic and/or pain-inducing substance through the epidermis into the dermis. A cascade of activation of histamine receptors, followed by itching and other reactions (neurogenic inflammation, oedema and erythema), is induced [21, 22].

Most of the respondents were farmers and agricultural activities generally take place during the rainy seasons when black fly densities are the highest. This might explain why more than half of the respondents reported having already stopped their activities because of the nuisance of black flies, which could lead to a reduction in their productivity as was previously observed in several other studies carried out in Nigeria [23–25]. There is therefore a need to implement vector control in the area, not only for onchocerciasis control but also to reduce black fly nuisance. Interviewees acknowledged that black flies bite more frequently during the rainy season, as higher monthly biting rates were previously reported in this area during this period [12, 26]. Altogether, this information provides indications for the ideal period for the implementation of any activity aiming at reducing vector densities and therefore their nuisance. Several respondents (59.1%) stated that black flies bite both outdoors and indoors, likely because of high densities of black flies in this area and the resulting strong competition for hosts. Only 10.7% of the participants were aware that fast flowing rivers constitute black fly breeding sites, while an important proportion (45.0%) thought that they breed in the forest on trees of the genus *Milicia*, commonly called Iroko, a tree widely distributed in Africa. This tree is in fact generally attacked at the leaf level by gall insects (mainly *Phytolyma lata*), which lay people can confuse with black flies. This inadequate knowledge of the bio-ecology of black flies, especially among populations living in communities where onchocerciasis is highly endemic, has also been previously documented [27, 28] and could be explained by the fact that in these areas actions regarding onchocerciasis always target parasites through MDA, while no vector control has been instituted and very limited information is provided on the latter apart from its role as a vector. This poor knowledge of the bio-ecology of black flies increases the population's exposure to bites and therefore to infection and could be a limitation of community activities trying to eliminate these vectors.

Despite > 20 years of CDTI and the many visits of research teams over the past 30 years in the surveyed communities, only 43.3% of the respondents had good knowledge of onchocerciasis and black fly bio-ecology. This could be explained by the fatigue observed during the CDD training (which remains just a formality) and their carrying out of the work. Indeed, in addition to the fact that the fight against onchocerciasis has been going on for a long time (and is still ongoing without clear prospects on a date to stop treatments), the CDDs are not very motivated because they work on a voluntary basis and therefore reduce their activities to the minimum, i.e. administering the drug without taking time for sensitisation/education of the population. In addition, the constant change of CDDs due to resignation or unavailability might also explain the poor knowledge of interviewees. A stronger sensitisation with diversified means and messages as well as discussion sessions could reinforce the knowledge of the population and optimise the results of the fight against this debilitating disease.

Wearing clothes covering the entire body as a means of protection against black fly bites has been reported in several studies conducted in many African countries [29, 30]. Although effective, this means of protection can be poorly tolerated at certain times of the day when the temperature exceeds 26 °C. The use of chemicals (especially gas oil) has also been reported in a study by Adeleke et al. in Nigeria [27]. Indeed, chemicals such as diesel have been used in combination with other chemicals as repellents for some insects and mammals [31, 32]. Although these substances can have the desired effect, it should be noted that they can be harmful when applied on the skin or inhaled (during application), leading to severe headaches, difficulty breathing, skin irritation or burns, etc. Similarly, lemon oil has been successfully used as a repellent against *Anopheles* and several other arthropods [33, 34]. However, applying raw lemon juice to the skin can also lead to lesions.

Almost all (95.3%) of the participants knew about ivermectin and 88.8% declared that they regularly swallow it during mass drug distributions. This important knowledge and adherence to ivermectin treatment are consistent with the study conducted in hyper-endemic communities in Nigeria [35]. This percentage is nevertheless higher than that reported by Kamga and colleagues in the Bafia Health District where 61.1% of the interviewees reported having taken the treatment in the last 5 years [36]. A few individuals reported having never swallowed ivermectin (11.2%) for a range of reasons, including fear of adverse reactions, during mass drug campaigns. This highlights the fact that some individuals are regularly untreated and can constitute a reservoir, thus showing the need for implementation of alternative or complementary strategies for onchocerciasis control in this area [37]. Most (78.6%) of the participants said that they would be ready to participate in the fight against black flies if an effective strategy were proposed to them, thus supporting the fact that, apart from being the vector of the *O. volvulus* parasite, black flies are a nuisance for the local populations. Indeed, community participation in onchocerciasis vector control has led to an important decrease in black fly densities in Mali and Uganda [13, 38]. Moreover, community participation can go beyond the mere involvement in activities, as some West African communities have even contributed financially to cover the costs of ground larviciding [39]. Such successful activities are dynamic processes based on strong partnerships between communities and local institutions [40].

According to the 2015 CDTI census, the population structure is male biased in Biatsota, though women are most represented in our sampling in this community; this is likely due to the door-to-door daytime survey approach we used since women are at home more during the day than males. In addition, the sample size was not reached as some households were closed during our daytime visit; this likely reduced the power to detect differences in knowledge and perception related to onchocerciasis and black fly bio-ecology and nuisance. However, it is worth mentioning that more than two-third of the households were surveyed in each of the surveyed communities. Finally, since other filarial diseases are endemic in the study area, it would have been interesting to add a specific question about onchocerciasis symptoms in the General Knowledge of Onchocerciasis section to avoid or limit any confusion about onchocerciasis and other filariases. However, we used local names of onchocerciasis and its vector to limit confusion.

## Conclusions

This study revealed that most of the respondents had already heard about onchocerciasis, even though the transmission mode and vector bio-ecology were not well known. The nuisance caused by black flies is a real concern for the population as it can seriously impact their economic productivity. These findings indicate an important opportunity to implement community-based vector control to both complement CDTI in the fight against onchocerciasis and reduce black fly nuisance. More efforts toward the education of local populations on black fly bio-ecology and preventive approaches and strong partnerships with communities and local institutions are required.

## Supplementary Information

**Additional file 1: Figure S1.** Map of Bafia Health District showing surveyed communities.

**Additional file 2: Table S1.** KAP survey form.

**Additional file 3: Table S2.** Details of the individuals who had never heard about onchocerciasis.

## Data Availability

All data generated or analysed during this study are included in this published article and its supplementary information files.

## Abbreviations

CDD: Community drug distributor
CDTI: Community-Directed Treatment with Ivermectin
KAP: Knowledge, attitude and practice
