# Supplementary figures and images for: Knowledge/perception and attitude/practices of populations of two first-line communities of the Centre Region of Cameroon regarding onchocerciasis and black fly nuisance and bio-ecology
Source: Parasit Vectors. 2021 Oct 23;14:546. doi: 10.1186/s13071-021-05048-y (PMC8542320; doi:10.1186/s13071-021-05048-y)

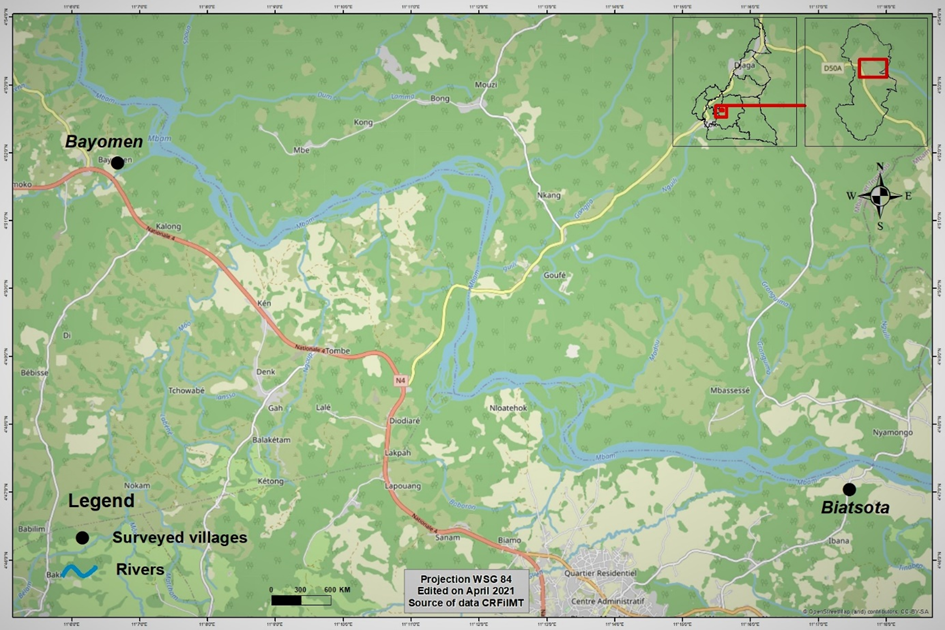


**Figure 1**: Map of the Bafia Health District showing surveyed communities

Supplement: Supplementary file 1 — Additional file 1: Figure S1. Map of Bafia Health District showing surveyed communities. [file 13071_2021_5048_MOESM1_ESM.docx]
