# Supplementary material for: Knowledge/perception and attitude/practices of populations of two first-line communities of the Centre Region of Cameroon regarding onchocerciasis and black fly nuisance and bio-ecology
Source: Parasit Vectors. 2021 Oct 23;14:546. doi: 10.1186/s13071-021-05048-y (PMC8542320; doi:10.1186/s13071-021-05048-y)
Supplement: Supplementary file 2 — Additional file 2: Table S1. KAP survey form. [file 13071_2021_5048_MOESM2_ESM.docx]

**KAP SURVEY FORM**

**Socio-demographic information**

Region: ___________ Health District: _______________ Health Area: _______________

Village: ________________________ Village code: |___| Date: |___|____|______|

N° order in village: |__|__|__| Final ID: |___|___|___|___|

Sex (M/F): |___| Age (official or stated): |____| years. Profession: __________________

Since how many years have you be living in the village: |_______| years?

Other place of residence before arrival in the village: _____________________________

**General Knowledge on onchocerciasis**

Have you ever heard of onchocerciasis? Yes |__| No |__|

If yes, do you know the transmission mode?

Blackfly bites |___| Mosquito’s bites |___| Dirty water |___|

**General knowledge on blackflies and its nuisance**

Have you ever heard of blackflies? Yes |__| No |__|

If yes, do you know that transmit diseases? Yes |__| No |__|

Do you know what disease they transmit? Yes |__| No |__|

If yes, which one? Malaria |__| Filaria |__| sleeping sickness |__| Scabies |__|

Do you often have post-biting sequels? Yes |__| Non |__|

If yes, what kind? Itching |___| Swelling |___| Pain |___|

Do blackflies often bother you at work? Yes |__| No |__|

Do blackflies bite you at home? Yes: |__| No |__|

If yes, indoor or outdoor? |___________________________________________|

Do you have any protection means? Yes |__| No |__|

If yes, which one? |___________________________________________|

During which period of the year are you most bitten by blackflies? |_________|

Do you know where blackflies breed? Yes: |__| No |__|

If yes, where? River |__| Bush |__| Grass |__| Stagnant water |__|

Can you help to control these flies? Yes: |__| No |__|.

**General knowledge and compliance to ivermectin treatment**

Do you know Mectizan? Yes |__| No |__|

Have you ever taken Mectizan? Yes |__| No |__|

When did you take it for the last take (year)? |____________|

Treatment compliance: 2019 |__| 2018 |__| 2017 |__| 2016 |__| 2015 |__| 2014 |__|.

If not, why? |__________________________________________________________|

**Remarks:** _________________________________________________________________
