# Supplementary material for: Knowledge/perception and attitude/practices of populations of two first-line communities of the Centre Region of Cameroon regarding onchocerciasis and black fly nuisance and bio-ecology
Source: Parasit Vectors. 2021 Oct 23;14:546. doi: 10.1186/s13071-021-05048-y (PMC8542320; doi:10.1186/s13071-021-05048-y)
Supplement: Supplementary file 3 — Additional file 3: Table S2. Details of the individuals who had never heard about onchocerciasis. [file 13071_2021_5048_MOESM3_ESM.docx]

**Details of the individuals who had never heard about onchocerciasis**

| Variables | No. individuals in Bayomen (%) | No. individuals in Biatsota | Total individuals visited (%) |
| --- | --- | --- | --- |
| Numbers | **13** | **12** | **25** |
| Age |  |  |  |
| [15-25] | 7 (28.0) | 4 (16.0) | 11 (44.0) |
| [26-35] | 2 (8.0) | 1 (4.0) | 3 (12.0) |
| [36-45] | 3 (12.0) | 3 (12.0) | 6 (24.0) |
| [46-55] | 1 (4.0) | 0 (0.0) | 1 (4.0) |
| > 55 | 0 (0.0) | 4 (16.0) | 4 (16.0) |
| Sex |  |  |  |
| Male | 6 (24.0) | 5 (20.0) | 11 (44.0) |
| Female | 7 (28.0) | 7 (28.0) | 14 (56.0) |
| Occupation |  |  |  |
| High risk occupation | 8 (32.0) | 5 (20.0) | 13(52.0) |
| Low risk occupation | 5 (28.0) | 7 (28.0) | 12 (48.0) |
| Years of residency |  |  |  |
| <1 | 1 (4.0) | 2 (8.0) | 3 (12.0) |
| [1-3] | 3 (12.0) | 0 (0.0) | 3 (12.0) |
| [4-6] | 2 (8.0) | 1 (4.0) | 3 (12.0) |
| [7-9] | 1 (4.0) | 0 (0.0) | 1 (4.0) |
| >10 | 6 (24.0) | 9 (37.5) | 15 (60.0) |
